# Speaking Tracheostomy Tube and Modified Mouthstick Stylus in a Ventilator-Dependent Patient with Spinal Cord Injury

**DOI:** 10.1155/2015/320357

**Published:** 2015-08-27

**Authors:** Eiji Mitate, Kensuke Kubota, Kenji Ueki, Rumi Inoue, Ryosuke Inoue, Kenta Momii, Hiroshi Sugimori, Yoshihiko Maehara, Seiji Nakamura

**Affiliations:** ^1^Section of Oral and Maxillofacial Oncology, Division of Maxillofacial Diagnostic and Surgical Sciences, Faculty of Dental Science, Kyushu University, 3-1-1 Maidashi, Higashi-ku, Fukuoka 812-8582, Japan; ^2^Emergency and Critical Care Center, Kyushu University Hospital, 3-1-1 Maidashi, Higashi-ku, Fukuoka 812-8582, Japan; ^3^Department of Integrated Therapy for Chronic Kidney Disease, Faculty of Medical Sciences, Kyushu University, 3-1-1 Maidashi, Higashi-ku, Fukuoka 812-8582, Japan; ^4^Special Patient Oral Care Unit, Kyushu University Hospital, 3-1-1 Maidashi, Higashi-ku, Fukuoka 812-8582, Japan; ^5^Cerebrovascular Center, Saga-Ken Medical Centre Koseikan, 400 Oaza Nakabaru, Kasemachi, Saga 840-8571, Japan

## Abstract

Communication is a serious problem for patients with ventilator-dependent tetraplegia. A 73-year-old man was presented at the emergency room in cardiopulmonary arrest after falling from a height of 2 m. After successful resuscitation, fractures of the cervical spine and cervical spinal cord injury were found. Due to paralysis of the respiratory muscles, a mechanical ventilator with a tracheostomy tube was required. First, a cuffed tracheostomy tube and a speaking tracheostomy tube were inserted, and humidified oxygen was introduced via the suction line. Using these tubes, the patient could produce speech sounds, but use was limited to 10 min due to discomfort. Second, a mouthstick stylus, fixed on a mouthpiece that fits over the maxillary teeth, was used. The patient used both a communication board and a touch screen device with this mouthstick stylus. The speaking tracheostomy tube and mouthstick stylus greatly improved his ability to communicate.

## 1. Introduction

The number of patients with traumatic spinal cord injury (SCI) has increased over the past three decades [1]. The incidence of SCI in Japan is 40.2 per million [2]. Patients with ventilator-dependent tetraplegia resulting from cervical SCI suffer from stress and medical problems. In particular, communication disorders cause frustration and anxiety. Approximately 1 in 5 patients with SCI have depression [3].

Many communication devices to assist patients with tetraplegia have been reported and available on the market [4, 5]. The speaking tracheostomy tube makes phonation possible, but it can only be used for a few minutes without discomfort. With a mouthstick stylus, patients can use a communication board and touchscreen devices, but they often become fatigued due to the need for constant biting. Some modification is needed.

In this paper, the efficacy of a speaking tracheostomy tube and “modified” mouthstick stylus for a patient with ventilator-dependent tetraplegia due to cervical SCI is reported.

## 2. Case Description and Results

**Figure 1:**
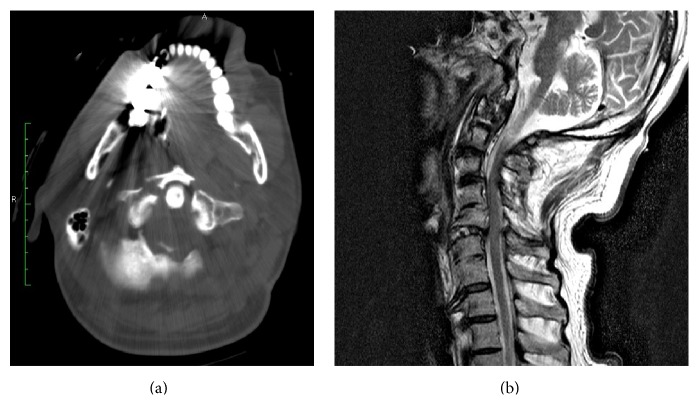
Computed tomography (CT) and magnetic resonance imaging (MRI) examination on arrival. (a) shows a Jefferson fracture. (b) shows a sagittal T2-weighted MRI of the cervical spinal cord injury (C1 and C2).

A 73-year-old male who fell from a height of 2 m was admitted to the hospital in cardiopulmonary arrest. His medical history included hypertension and urolithiasis. After successful resuscitation, fractures of the cervical spine (C1, C5, C6, and C7) and cervical SCI (C1 and C2) were found on examination by computed tomography (CT) and magnetic resonance imaging (MRI) (Figure 1). He became tetraplegic and required mechanical ventilator support. He underwent a tracheotomy with a cuffed tracheostomy tube (Blue Line Profile Cuff; Smiths Medical, Inc.), and soon had difficulty with communication and became depressed. First, he spoke in a hoarse whisper, assisted with 5 L/min of humidified oxygen gas via the suction line. This method could only be used for a few minutes and caused discomfort. Second, a speaking tracheostomy tube (Vocalaid; Smiths Medical, Inc.) was inserted. He could talk for up to 10 min using this tube, but with fatigue. Moreover, speaking with the tracheostomy tube was inadequate for communication.

**Figure 2:**
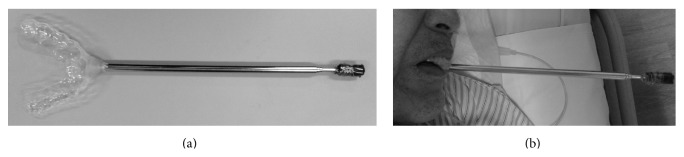
Modified mouthstick stylus. (a) shows an overview of the modified mouthstick stylus. (b) shows the mouthstick stylus fixed on the maxillary teeth. The patient can use this stylus without biting.

The mouthstick stylus is also commercially available. However, the constant biting required for its operation left him fatigued. Thus, a stylus fixed on a mouthpiece of maxillary teeth with dental self-curing resin (UNIFAST III; GC Dental, Japan) was applied. The mouthpiece was made with a 2 mm thick polyethylene terephthalate glycol-modified thermoforming plate (Erkodur; ERKODENT Erich Kopp GmbH, Germany). The stylus was made with a rod antenna and conductive urethane foam for electrostatic discharge (ESD form F-10-A; Hozan Tool Ind. Co., Ltd., Japan) (Figure 2). This modified mouthstick stylus was fixed on the maxilla without the need for biting and could be used with both a communication board and a capacitive iPad touchscreen. The length of the stylus was adjustable. As this provided the patient with a method for communication, he can communicate with us.

## 3. Discussion

SCI prevention should focus on sports and motorcycle accidents involving young people, traffic accidents involving adults, and falling accidents involving aged people [2]. Traumatic SCI drastically affects the quality of life. In particular, cervical SCI may cause tetraplegia, which requires a tracheostomy with a mechanical ventilator.

Our modified mouthstick stylus firmly fixed on the maxillary teeth can be used for communication boards, keyboards, and capacitive touchscreen interfaces such as the iPhone and iPad with little risk of being dropped. 69.2% of patients with SCI use a computer; of those, 94.2% access the Internet [6]. Many assistive devices for patients with SCI are currently available, one of which is the mouthstick stylus [7]. Our stylus will be of great help to patients with tetraplegia.

The speaking tracheostomy tube for patients with tetraplegia was first reported in 1967 and it has been in routine use since then [8]. Warm and humidified oxygen or air passing the vocal cords via a suction catheter makes a hoarse whisper only for a few minutes.

The speaking tracheostomy tube has some advantages. Continuous oxygen insufflation prevents aspiration during feeding, airway obstruction, and pulmonary infection [9]. On the other hand, there are some disadvantages such as pain, discomfort, and emphysema reported [10, 11]. Oxygen misconnected to the tracheostomy cuff punctured the cuff [12]. All locations of potential tube must be checked to protect against the adverse events.
